# Supplementary material for: Mechanical Brain Injury Increases Cells’ Production of Cystathionine β-Synthase and Glutamine Synthetase, but Reduces Pax2 Expression in the Telencephalon of Juvenile Chum Salmon, Oncorhynchus keta
Source: Int J Mol Sci. 2021 Jan 28;22(3):1279. doi: 10.3390/ijms22031279 (PMC7865298; doi:10.3390/ijms22031279)
Supplement: Supplementary file 1 [file ijms-22-01279-s001.pdf]

Table S1. Morphometric characteristics of glutamine synthetase-positive cells (M ± SD) in the pallial and subpallial zones of the telencephalon in intact juvenile chum salmon *Oncorhynchus keta* and on day 3 days post-injury

Pallial/subpallial zone	Size of GS labeled cells (μm), area of localization, intensity of immunolabeling					
	**Intact animals**			**Injured telencephalon**		
	Intense labeling	***Moderate labeling***	***Negative***	***Intense labeling***	***Moderate labeling***	***Negative***
**DD**	4.5±0.5/4.3±0.3 (PVZ)<br>7.8±0.7/6.3±0.6 (PVZ, PZ)	4.8±1.0/3.6±0.6 (SVZ)<br>9.5±1.4/7.1±0.8 (PVZ, PZ)	10.1±1.0/7.4 ±0.8 (PVZ, SVZ)<br>7.2±0.5/5.7±0.5 (SVZ, PZ)	4.5±0.5/4.3±0.3 (PVZ)<br>7.8±0.9/5.5±0.9* (DD1, DD2 PVZ)<br>8.1±0.6/6.2±0.5 (DD1, DD2, DD3 PVZ)<br>7.8±0.8/6.3±0.4* (DD3 PVZ	7.3±0.9/5.7±0.9 (DD1, DD2, PVZ, SVZ)<br>7.8±0.6/5.9±0.5(DD3, SVZ)	5.7±1.8/4.3±1.7 (PVZ)<br>7.5±1.1/6.0±1.1(DD1, DD2, DD3 SVZ, PZ)<br>8.2±0.9/4.2±0.2 (DD1, DD2, DD3, PZ)
**DM**	1.5±0.2/1.2±0.1 (PVZ, SVZ, PZ)<br>4.4±0.5/4.2±0.3 (PVZ, SVZ)<br>7.6±0.8/7.3 ±0.6 (PVZ, SVZ, PZ)	1.3±0.2/1.1±0.1 (SVZ, PZ)<br>4.6±0.3/4.4±0.2 (SVZ)<br>8.6±0.6/7.0 ±0.7 (SVZ, PZ)	9.3±0.8/6.8±1.0 (PVZ, SVZ)<br>8.3±0.4/4.8±0.6 (PZ)	4.6±0.5/4.2±0.4(PVZ)<br>7.8±0.9/5.5±0.9* (DM1, DM2, DM3 PVZ)<br>8.3±0.5/5.8±0.8 (DM1, DM2, DM3 PVZ)	4.5±0.5/4.3±0.3 (DM1, DM2, DM3 PVZ)<br>7.2±0.8/5.5 ±0.7* (DM1, DM2, DM3 PVZ, SVZ)	7.2±0.5/6.2±0.9 (PVZ)<br>7.7±0.4/4.5± 0.5 (DM1, DM2, DM3, SVZ, PZ).
**DL**	4.5±0.5/4.3±0.3 (PVZ)<br>7.9±0.6/4.2±0.6 (PVZ, PZ)	4.4±0.4/4.3±0.5 (PVZ, PZ)<br>7.7±0.6/6.4±0.5(PZ)	5.7±0.4/4.4±0.2 (PVZ, PZ)<br>8.4±0.6/5.8±0.9 (PZ)	7.0±0.2/4.2±0.2 (DL1, DL2, DL3 PVZ, DL2 SVZ)<br>8.1±0.7/5.7±0.6* (DL1, DL2, DL3 PVZ)	4.7±0.3/4.4±0.2 (DL2, DL3 PVZ)<br>6.8±0.3/4.3±0.2 (DL1, DL2, SVZ)	6.9±0.4/6.3±0.4 (PVZ)<br>7.4±0.7/4.7±0.5 (DL1, DL2, DL3 SVZ, PZ)
**VD**	4.8±0.8/3.6±0.5 (PVZ)<br>7.2±0.4/6.3±0.3 (PVZ, PZ)	1.1±0.2/0.9±0.2 (PVZ, SVZ, PZ)<br>4.7±0.4/4.5±0.3 (SVZ, PZ)<br>8.2±0.5/7.0 ±0.4 (SVZ, PZ)	7.4±1.0/5.2±1.2 (PVZ)<br>8.5±0.3/6.4±0.4 (SVZ, PZ).	4.5±0.5/3.7±0.3 (VD1, VD2 PVZ)<br>9.3±0.7/3.8±0.3 (VD1 PVZ)<br>7.8±0.5/5.8±0.5* (VD1, VD2 PVZ)	4.6±0.4/3.7±0.4 (VD1, VD2 PVZ)<br>8.7±0.7/4.1±0.3 (VD2 SVZ)	6.5±0.3/5.5±0.3(VD1, VD2 PVZ, SVZ)<br>9.5±0.5/3.7±0.4 (VD1 PVZ)<br>8.2±0.4/5.7±0.5 (VD1, VD2, PZ)
**VV**	1.3±0.3/1.2±0.3 (SVZ)<br>5.6±0.2/2.8±0.2 (PVZ)<br>7.4±0.5/6.6±0.4 (PVZ, PZ)	1.2±0.1/0.9±0.3 (PVZ, SVZ, PZ)<br>5.2±0.3/4.6±0.4 (PVZ, SVZ, PZ)<br>7.2±0.3/6.4±0.5 (PVZ, SVZ, PZ)	7.2±0.5/5.7±0.2 (SVZ, PZ)<br>8.4±0.7/6.4±0.5 (SVZ, PZ).	4.4±0.6/3.8±0.3 (VV2 PVZ)<br>8.3±0.5/5.8±0.6 (VV1 PVZ)<br>7.8±0.5/5.8±0.5* (VV1, PVZ)	7.8±0.6/6.1±0.6 (VV1 SVZ, PZ)	7.2±0.8/5.3±0.7 (VV1, SVZ)<br>6.8±0.5/5.5±0.7 (VV2, SVZ, PZ)
**VL**	5.1±0.8/4.1±0.9 (PVZ)	1.3±0.2/1.0±0.3 (PVZ, SVZ, PZ)	5.2±0.3/4.7±0.3 (PZ)<br>6.4±0.3/6.1±0.2 (SVZ,	5.1±0.5/3.9±1.1 (VL1, VL2, PZ)	4.5±0.3/3.7±0.2 (VL1, SVZ)	8.7±0.6/6.0±0.3 (VL1, VL2, SVZ, PZ)

	7.6±0.3/6.8±0.2 (PZ)	5.3±0.3/4.5±0.4 (SVZ. PZ)<br>7.4±0.5/6.5±0.3 (PZ)	PZ)		6.5±0.3/4.4±0.4 (VL2, SVZ)<br>8.1±0.5/5.4±0.5* (VL1, PVZ)	

Large- and small-sizes of neurons are shown through a slash. Cells were morphologically classified according to a previously developed scheme [6]. * indicates radial glia that appear in the intact juvenile chum salmon *O. keta* telencephalon and/or after injury.

Table S2. Morphometric characteristics of cystathionine β-synthase (CBS) positive cells (M ± SD) in the pallial and subpallial zones of the telencephalon in intact juvenile chum salmon *Oncorhynchus keta* and on day 3 post-injury

Pallial/subpallial zone	**Size of CBS-labeled cells (µm), area of localization, intensity of immunolabeling**					
	**Intact animals**			**Injured telencephalon**		
	Intense labeling	***Moderate labeling***	***Negative***	***Intense labeling***	***Moderate labeling***	***Negative***
**DD**	4.6±0.6/4.4±0.3 (PVZ)<br>6.7±0.7/6.3±0.8 (PVZ, SVZ)	1.3±0.1/1.0±0.2 (PVZ, SVZ, PZ)<br>4.5±0.5/4.3±0.4 (SVZ)<br>9.5±1.4/7.1±0.8 (PVZ, PZ)	10.1±1.0/7.4 ±0.8 (PVZ, SVZ)<br>7.6±0.9/6.5±0.7 (SVZ, PZ)	6.7±0.3/5.1±0.6 (PVZ, SVZ, PZ)<br>8.1±0.6/6.2±0.5 (PVZ, PZ)	7.3±0.9/5.7±0.9 (SVZ, PZ)	8.1±0.5/6.6±0.5 (SVZ, PZ)
**DM**	4.4±0.5/4.2±0.3 (PVZ, SVZ)<br>7.8±0.7/7.3 ±0.6 (PVZ, SVZ, PZ)	1.4±0.2/1.1±0.1 (PVZ, SVZ, PZ)<br>9.2±0.9/7.0 ±0.7 (SVZ, PZ)	9.3±0.8/6.8±1.0 (PVZ, SVZ)<br>6.3±0.4/3.8±0.6 (PZ)	5.9±0.9/4.8±0.5 (PVZ, SVZ)<br>7.8±0.5/5.8±0.7 (PZ)	5.5±0.5/4.7±0.3 (PVZ, SVZ)<br>8.0±0.4/5.9±0.4 (PZ)	8.1±0.5/6.2±0.9 (SVZ, PZ).
**DL**	4.4±0.5/4.3±0.2 (PVZ)<br>8.0±1.4/6.1±1.0 (PVZ, PZ)	4.4±0.4/4.3±0.5 (PVZ)<br>8.1±0.3/6.1±0.7 (PZ)	5.7±0.4/4.4±0.2 (PVZ, PZ)<br>8.8±1.3/6.5±0.7 (PZ)	5.6±0.7/5.1±0.4 (PVZ)<br>7.8±0.7/5.5±0.4 (PZ)	5.7±0.8/4.4±0.3 (SVZ, PZ)	6.9±0.8/5.4±0.9 (SVZ, PZ)<br>8.9±0.7/7.2±0.7 (PZ)
**VD**	1.2±0.1/1.0±0.3 (PVZ)<br>4.8±0.8/3.6±0.5 (PVZ)<br>8.0±0.8/6.5±0.7 (PVZ,PZ)	1.1±0.2/0.9±0.2 (PVZ, SVZ)<br>8.1±0.8/6.5±0.5 (PZ)	7.4±1.0/5.2±1.2 (PVZ)<br>6.3±0.4/3.8±0.6 (SVZ, PZ)<br>8.4±0.9/6.1±0.9 (SVZ, PZ)	6.6±0.6/5.6±0.5 (PVZ, SVZ, PZ)	6.4±0.8/5.8±0.5 (PVZ, SVZ, PZ)<br>7.4±0.3/6.4±0.3 (PZ)	7.8±0.4/5.3±0.4 (PVZ, SVZ)<br>8.2±0.4/6.1±0.3(PZ)
**VV**	6.6±0.3/3.6±0.4 (PVZ)<br>7.4±0.5/6.6±0.4 (PVZ, PZ)	1.2±0.2/1.1±0.3 (PVZ, SVZ, PZ)<br>5.2±0.3/4.6±0.4 (PVZ, SVZ, PZ)<br>7.6±0.7/5.7±0.7 (SVZ,	7.4±0.4/5.6±0.4 (SVZ, PZ)	4.7±0.2/3.4±0.4 (PVZ)<br>8.3±0.5/3.8±0.6 (PVZ)<br>7.3±0.4/6.3±0.3 (PZ)	6.2±0.6/5.7±0.3 (PVZ, SVZ, PZ)	6.8±0.5/5.5±0.7 (SVZ, PZ)<br>7.2±0.3/5.3±0.7 (SVZ)

		PZ)				
**VL**	4.4±0.8/4.1±0.6 (PVZ, SVZ, PZ)<br>9.0±0.3/7.3±0.7 (PZ)	1.3±0.2/1.0±0.3 (PVZ)<br>5.3±0.3/4.5±0.4 (PVZ, SVZ)<br>8.5±0.7/6.1±0.4 (PZ)	5.2±0.3/4.7±0.3 (PZ)<br>8.6±0.3/6.8±0.7 (SVZ, PZ)	5.8±0.7/6.3±0.7 (PVZ, SVZ, PZ)	5.8±0.4/5.7±0.4 (PVZ, SVZ)<br>6.5±0.3/4.4±0.4 (SVZ, PZ)	5.8±0.7/6.3±0.9 (SVZ, PZ)

Table S3. Morphometric characteristics of Pax2 expressing cells (M ± SD) in the pallial and subpallial zones of the telencephalon of intact juvenile chum salmon *Oncorhynchus keta* and on day 3 post-injury.

Pallial/ subpallial area	**Size of Pax2-expressing cells (μm), area of localization, intensity of immunolabeling**							
	**Intact animals**				**Injured telencephalon**			
	Intense labeling	***Moderate labeling***	***Weak labeling***	***Negative***	***Intense labeling***	***Moderate labeling***	***Weak labeling***	***Negative***
**DD**	4.2±0.8/2.7±0.4 (PVZ, nuclei)<br>5.7±0.7/5.3±0.7 (PVZ, SVZ)	3.5±0.5/3.3±0.2 (SVZ, PZ, nuclei)<br>9.5±1.4/7.1±0.8 (PVZ, PZ)	4.8±0.3/4.2±0.3 (SVZ, PZ)	5.7±0.9/4.3±0.7 (PZ)<br>7.9±0.6/6.7±0.5 (PZ)	3.3±0.5/3.1±0.3 (PVZ, SVZ, PZ. nuclei)<br>6.5±0.4/2.8±0.4 (PVZ)<br>5.5±1.4/6.1±0.4 (PVZ, PZ)	3.2±0.4/2.9±0.3 (PVZ, SVZ, PZ. nuclei)<br>5.9±0.6/5.7±0.3 (SVZ, PZ)	-	6.3±0.5/5.6±0.5 (PVZ, SVZ, PZ)<br>8.0±0.7/6.7±0.7 (PZ)
**DM**	3.5±0.5/2.7±0.3 (PVZ, SVZ, nuclei)<br>7.8±0.7/7.3 ±0.6 (PVZ, SVZ, PZ)	1.4±0.2/1.1±0.1 (SVZ, PZ)<br>3.4±0.4/2.7±0.3 (PVZ, SVZ, nuclei)<br>5.0±0.4/3.7±0.6 (SVZ, PZ)	5.2±0.4/4.5±0.4 (PZ)	6.6±1.0/4.8±0.5 (SVZ, PZ)<br>9.1±1.0/4.4 ±0.6 (PZ)	3.1±0.3/2.9±0.2 (PVZ,SVZ, nuclei)<br>6.5±0.4/2.8±0.3 (PVZ)<br>5.4±0.6/5.2±0.6 (PVZ, SVZ)	3.1±0.3/2.9±0.3 (PVZ, SVZ, nuclei)<br>5.5±0.5/4.7±0.3 (SVZ, PZ)	6.8±0.8/5.1±0.3 (SVZ. PZ)	7.2±0.4/5.4±0.5 (PVZ. SVZ)<br>8.2±0.6/6.8±0.5 (PZ)
**DL**	3.4±0.4/2.6±0.4 (PVZ, nuclei)<br>4.8±0.7/3.8±0.3 (PVZ, SVZ)<br>8.8±0.6/6.1±1.0 (SVZ, PZ)	4.4±0.4/4.3±0.5 (PVZ)<br>6.1±0.3/5.5±0.7 (SVZ, PZ)	5.7±0.4/5.1±0.4 (PZ)	6.4±0.6/4.7±0.4 (SVZ, PZ)<br>8.7±0.9/4.2 ±0.7 (PZ)	4.1±0.3/6.1±0.6 (PVZ, SVZ)	3.2±0.3/3.0±0.3 (PVZ, SVZ, nuclei)<br>5.7±0.8/4.4±0.3 (SVZ, PZ)	6.6±0.7/5.2±0.3 (SVZ, PZ)	5.6±0.4/4.4±0.3 (PVZ, SVZ)<br>7.2±0.8/5.8±0.7 (SVZ, PZ)
**VD**	4.4±0.8/3.0±0.4 (PVZ)	3.4±0.3/2.7±0.3 (SVZ, PZ)<br>8.2±0.7/6.5±0.5	6.7±0.5/5.2±0.3 (PZ)	5.6±1.1/3.7±0.8 (PVZ, SVZ)<br>7.9±0.6/6.7±0.5 (PZ)	3.1±0.3/3.1±0.3 (PVZ, nuclei)<br>4.1±0.4/5.5±1.1	3.2±0.2/2.9±0.3 (PVZ, SVZ, nuclei)	5.8±0.4/4.8±0.4 (SVZ, PZ)	6.4±1.0/5.2±1.2 (PVZ, SVZ)<br>7.5±0.8/6.1±0.3

		(SVZ, PZ)		8.9±0.9/3.9 ±0.6 (PZ)	(PVZ, SVZ)	4.8±0.4/4.2±0.5 (SVZ, PZ)		(SVZ, PZ)
**VV**	5.0±0.9/3.8±0.6 (PVZ, SVZ)	3.4±0.4/2.7±0.3 (PVZ, SVZ, nuclei)<br>7.6±0.7/5.7±0.7 (SVZ, PZ)	6.4±0.7/5.3±0.6 (PZ)	6.2±0.9/4.3±1.11 (SVZ, PZ)<br>7.7±0.6/6.8±0.4 (PZ)<br>9.1±0.9/3.9 ±0.2 (PZ)	3.1±0.2/2.9±0.3 (PVZ, nuclei)<br>6.6±1.0/5.6±0.6 (PVZ)<br>7.2±0.4/6.3±0.3 (PZ)	3.1±0.2/2.9±0.3 (PVZ, nuclei)<br>6.2±0.6/5.7±0.3 (SVZ, PZ)	5.6±0.3/4.8±0.4 (SVZ, PZ)	6.2±1.3/5.6±0.3 (PVZ, SVZ, PZ)
**VL**	3.5±0.3/2.7±0.3 (PVZ, SVZ nuclei)<br>6.2±0.5/4.1±0.5 (PZ)	3.5±0.3/2.7±0.3 (PVZ, SVZ nuclei)<br>5.4±1.0/3.9±0.6 (SVZ, PZ)	6.2±0.5/4.8±0.4 (PZ)	6.5±1.2/4.6±0.3(SVZ, PZ)<br>7.9±0.7/6.6±0.5 (PZ)<br>8.9±0.9/4.0 ±0.7 (PZ)	3.2±0.3/3.0±0.2 (PVZ, nuclei)<br>6.1±0.4/4.5±0.6 (PVZ, SVZ)	3.1±0.2/3.0±0.2 (PVZ, SVZ nuclei)<br>5.8±0.3/5.3±0.4 (SVZ)<br>6.5±0.5/4.6±0.4 (SVZ, PZ)	5.5±0.4/4.8±0.6 (PZ)	5.2±1.1/4.6±1.4 (PVZ, SVZ, PZ)
